# Imipenem represses CRISPR-Cas interference of DNA acquisition through H-NS stimulation in *Klebsiella pneumoniae*

**DOI:** 10.1038/srep31644

**Published:** 2016-08-17

**Authors:** Tzu-Lung Lin, Yi-Jiun Pan, Pei-Fang Hsieh, Chun-Ru Hsu, Meng-Chuan Wu, Jin-Town Wang

**Affiliations:** 1 Department of Microbiology, National Taiwan University College of Medicine, Taipei, Taiwan; 2 Department of Microbiology, School of Medicine, China Medical University, Taichung, Taiwan; 3 Department of Medical Research, E-Da Hospital, Kaohsiung, Taiwan; 4 School of Medicine, I-Shou University, Kaohsiung, Taiwan; 5 Department of Internal Medicine, National Taiwan University Hospital, Taipei, Taiwan

## Abstract

Analysis of the genome of *Klebsiella pneumoniae* NTUH-K2044 strain revealed the presence of two clustered regularly interspaced short palindromic repeats (CRISPR) arrays separated with CRISPR-associated (*cas*) genes. Carbapenem-resistant *K. pneumoniae* isolates were observed to be less likely to have CRISPR-Cas than sensitive strains (5/85 vs. 22/132). Removal of the transcriptional repressor, H-NS, was shown to prevent the transformation of plasmids carrying a spacer and putative proto-spacer adjacent motif (PAM). The CRISPR-Cas system also decreased pUC-4K plasmid stability, resulting in plasmid loss from the bacteria with acquisition of new spacers. Analysis of the acquired proto-spacers in pUC-4K indicated that 5′-TTN-3′ was the preferred PAM in *K. pneumoniae*. Treatment of cells by imipenem induced *hns* expression, thereby decreasing *cas3* expression and consequently repressed CRISPR-Cas activity resulted in increase of plasmid stability. In conclusion, NTUH-K2044 CRISPR-Cas contributes to decrease of plasmid transformation and stability. Through repression of CRISPR-Cas activity by induced H-NS, bacteria might be more able to acquire DNA to confront the challenge of imipenem.

Arrays of clustered regularly interspaced short palindromic repeats (CRISPRs) are widespread in the genome of many bacteria and almost all archaea. CRISPRs are composed of direct repeats which are separated by similar-sized spacer sequences^1^,^2^. Accompanying CRISPRs, there are approximately four to ten CRISPR-associated (*cas*) genes. Spacer sequences are reported to be similar to those of plasmid or phage DNA. Therefore, CRISPRs were proposed to be a defense mechanism against infection by extra-chromosomal agents. Recent studies discovered that, in response to phage infection, bacteria can integrate phage genomic sequences as new spacers in CRISPR^3^ and thereby block subsequent phage infections. The removal of these spacers resulted in loss of resistance^3^. The repeat-spacer array can be transcribed and then processed as small RNAs with the participation of Cas proteins that base-pair with phage nucleic acids, leading to their degradation^4^. The CRISPR-Cas system was also shown to cleave plasmid DNA. Such “immunity” protects bacteria from phage infection and other horizontal gene transfer^5^,^6^,^7^.

Antibiotic resistance is often mediated by acquisition of resistance genes frequently located on mobile elements including plasmids. The correlation between antibiotic resistance and the presence of CRISPR-Cas has recently been studied. A highly significant inverse correlation between the presence of CRISPR-Cas loci and acquired antibiotic resistance was revealed in 48 *Enterococcus faecalis* strains^8^. However, no association between *cas* genes and antibiotic resistance was observed in 263 natural *Escherichia coli* strains^9^.

*Klebsiella pneumoniae* is an important human pathogen both in hospital or community settings. Increasing resistance to antibiotics such as extended-spectrum β-lactams and carbapenem in *K. pneumoniae* is a significant problem. Acquisition of genes encoding antibiotic hydrolysis enzymes such as extended-spectrum β-lactamase (ESBL) and carbapenemase contribute to the resistance. Analysis of the full genome sequence of *K. pneumoniae* NTUH-K2044 strain revealed two putative CRISPR arrays. Therefore, we studied the structure, function, and regulation of CRISPR-Cas systems, and the correlation between CRISPR-Cas systems and antibiotic resistance in *K. pneumoniae*.

## Results

### Analysis of CRISPR-Cas system in *K. pneumoniae* NTUH-K2044 strain

**Figure 1:**
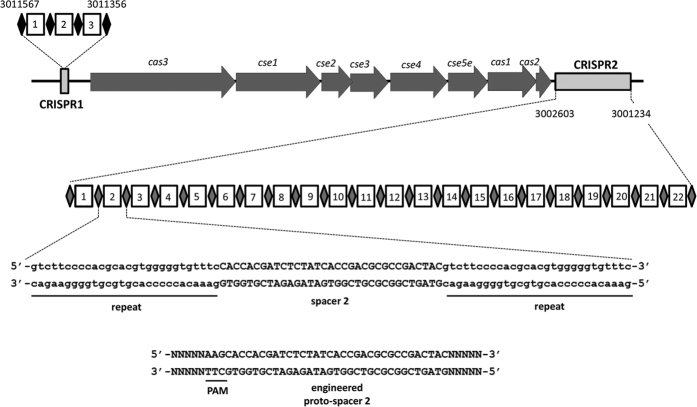
The structure of CRISPR-Cas system in *K. pneumoniae* NTUH-K2044 strain. A *cas* locus located between two CRISPR arrays (CRISPR1 and CRISPR2) in *K. pneumoniae* NTUH-K2044 strain was revealed. CRISPR1 array composed of four repeated sequences (solid diamond) and three spacer sequences (open square) was located at nucleotides 3011356 to 3011567. CRISPR2 array composed of twenty-three repeated sequences and twenty-two spacer sequences was located at nucleotides 3001234 to 3002603. Sequences of spacer 2 flanked by repeat sequences in CRISPR2 and engineered proto-spacer 2 (sequences matching spacer 2 and putative PAM) are shown.

Analysis of the full genome sequence of *K. pneumoniae* NTUH-K2044 strain (accession numbers of the chromosome and plasmid sequences: AP006725 and AP006726, respectively) revealed two putative CRISPR arrays. One CRISPR array (known as CRISPR1) was composed of four 29-bp repeat sequences and three spacer sequences located at nucleotides 3011356 to 3011567 (Fig. 1). The other CRISPR array (known as CRISPR2) consisted of twenty-three 28-bp repeat sequences and twenty-two spacer sequences located at nucleotides 3001234 to 3002603 (Fig. 1). Sequences between CRISPR1 and CRISPR2 were identified as CRISPR-associated (*cas*) genes including *cas2, cas1, cse5e, cse4, cse3, cse2, cse1*, and *cas3*. Therefore, the alignment of CRISPR-Cas in *K. pneumoniae* NTUH-K2044 strain was similar to that of the type I-E CRISPR-Cas in *E. coli*^1^.

### Prevalence of CRISPR-Cas among drug resistant and sensitive strains

CRISPR-Cas was demonstrated to interfere with the transformation and stability of plasmids that often carry drug resistant genes^5^,^6^,^7^. Accordingly, we assessed whether presence of CRISPR-Cas in clinical strains is related to their drug resistance. PCR using *cas1*, CRISPR1, and CRISPR2 primers found that all of the *cas1* PCR positive strains also had at least one CRISPR array (CRISPR1 or CRISPR2). The prevalence of CRISPR-Cas system was 5/85 in carbapenem-resistant strains and 22/132 in drug-sensitive intestinal strains, showing a highly significant inverse correlation between prevalence and resistance (*P* = 0.0205, chi-square test). The low frequency of CRISPR-Cas system in drug resistant *K. pneumoniae* implied that CRISPR-Cas may play a role in preventing acquisition of drug resistance genes.

### The function of NTUH-K2044 CRISPR-Cas in plasmid transformation

**Figure 2:**
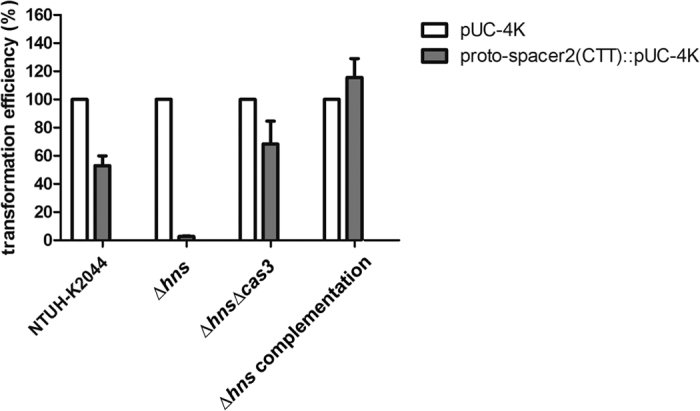
The transformation efficiencies of pUC-4K and pUC-4K with engineered proto-spacer 2 identical with spacer 2 of CRISPR2 in NTUH-K2044 wild type, *hns* deletion (∆*hns*), *hns* and *cas3* double deletion (∆*hns*∆*cas3*) and *hns* complementation strains. The transformation efficiency of pUC-4K was set as 100% and that of proto-spacer2(CTT)::pUC-4K was calculated accordingly. Data are presented as means ± SEM from three independent experiments.

The CRISPR-Cas system was reported to be involved in resistance to plasmid transformation^5^,^6^,^7^. Recent studies reveal that the recognition of proto-spacer adjacent motif (PAM) is essential for the targeting of CRISPR-Cas^10^. Therefore, an engineered proto-spacer containing spacer sequences identical to spacer 2 (Fig. 1) in the NTUH-K2044 CRISPR2 and the predicted PAM sequence (5′-CTT-3′) of type I-E CRISPR-Cas^11^,^12^ were cloned into a pUC-4K plasmid. Then, the transformation efficiencies of plasmids with or without engineered proto-spacer sequences were compared. Inserting proto-spacer sequences into a pUC-4K plasmid significantly decreased the transformation efficiencies in wild type strain by approximately 50% (Fig. 2).

A histone-like DNA-binding protein that can modulate gene expression globally, H-NS has been shown to repress the expression of CRISPR-Cas in *E. coli*^13^,^14^,^15^. Therefore, we examined whether H-NS represses the CRISPR-Cas in *K. pneumoniae* NTUH-K2044 strain. Deletion of *hns* revealed a tenfold increase in the absolute transformation efficiency with a pUC-4K plasmid (data not shown). The transformation efficiency of a plasmid with an added proto-spacer sequence was significantly decreased to approximately 3% of that of a control plasmid in NTUH-K2044 *hns* deletion (∆*hns*) mutant (Fig. 2). The *cas3* gene encoding a DNA nuclease/helicase responsible for degradation of targets in CRISPR interference^1^,^2^ was further deleted in ∆*hns* mutant to determine whether CRISPR-Cas interfered with transformation in the ∆*hns* mutant. Deletion of *cas3* significantly restored the transformation efficiency in ∆*hns* mutant, of the plasmids carrying proto-spacer sequences (Fig. 2). Therefore, in the absence of the CRISPR-Cas repressor, H-NS, CRISPR-Cas in NTUH-K2044 could prevent the transformation of plasmids carrying spacer sequences identical to those of CRISPR.

The *hns* complementation strain was also generated to further confirm the repression effect of H-NS on CRISPR-Cas activity. No interference with transformation of plasmids carrying sequences identical to those of CRISPR spacers was apparent in the *hns* complementation strain (Fig. 2).

### H-NS repressed CRISPR-Cas RNA expression

**Figure 3:**
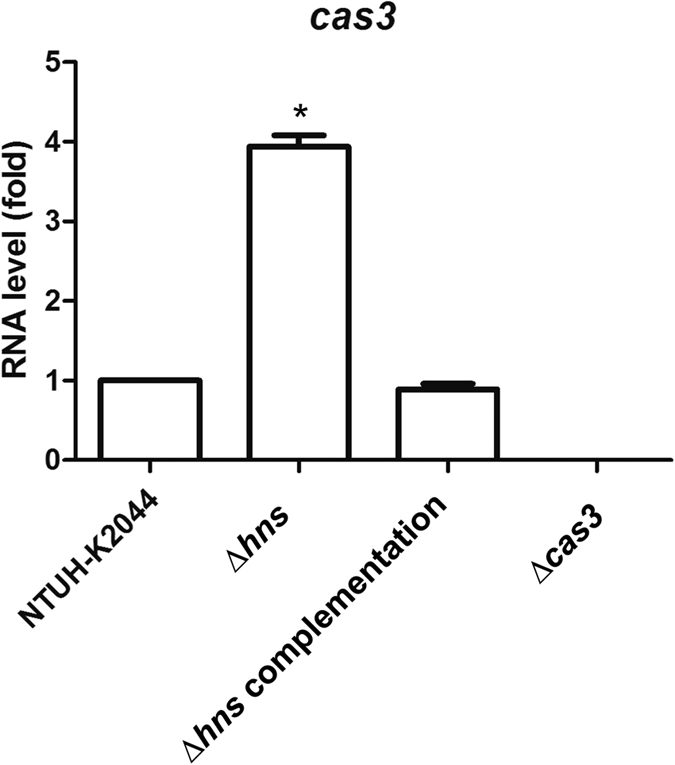
The RNA level of *cas3* in NTUH-K2044 wild type, *hns* deletion (∆*hns*), *hns* complementation and *cas3* deletion (∆*cas3*) strains. The RNA level in NTUH-K2044 was set as 1, and those in ∆*hns, hns* complementation and ∆*cas3* strains were calculated accordingly. Data are presented as means ± SEM from three independent experiments. *P-values of <0.05 were considered significant (Student’s *t*-test).

The expression of *cas3* RNA in the NTUH-K2044 wild type, ∆*hns* mutant, and *hns* complementation strains was determined by quantitative RT-PCR. The expression of *cas3* RNA was significantly increased by deletion in the ∆*hns* mutant and restored to wild-type levels in the *hns* complementation strain (Fig. 3). Our results in line with a previous study^16^ confirmed that H-NS could repress the expression of CRISPR-Cas.

### The function of NTUH-K2044 CRISPR-Cas system in phage resistance

The CRISPR-Cas system was reported to be involved in phage resistance^3^,^4^,^5^. In our previous study, we isolated a bacteriophage, NTUH-K2044-K1-1, that infects *K. pneumoniae* NTUH-K2044^17^. In order to test the function of NTUH-K2044 CRISPR-Cas system in phage resistance, clones of NTUH-K2044 ∆*hns* mutant (with de-repressed expression of CRISPR-Cas) showing resistance to phage NTUH-K2044-K1-1 infection were selected after overnight co-culture of bacteria and phage. Consistent with the finding that capsules are essential for infection by this phage, the majority of the resistant clones were found to be non-encapsulated. Therefore, the non-encapsulated variants are not expected to have acquired new spacers and were not tested further. We identified 427 encapsulated clones after screening of approximately one hundred thousand resistant-clones and tested those for new spacers. However, no new spacers were found. Therefore, CRISPR-Cas seemed not to be involved in resistance to phage NTUH-K2044-K1-1.

### The function of NTUH-K2044 CRISPR-Cas in plasmid stability

**Figure 4:**
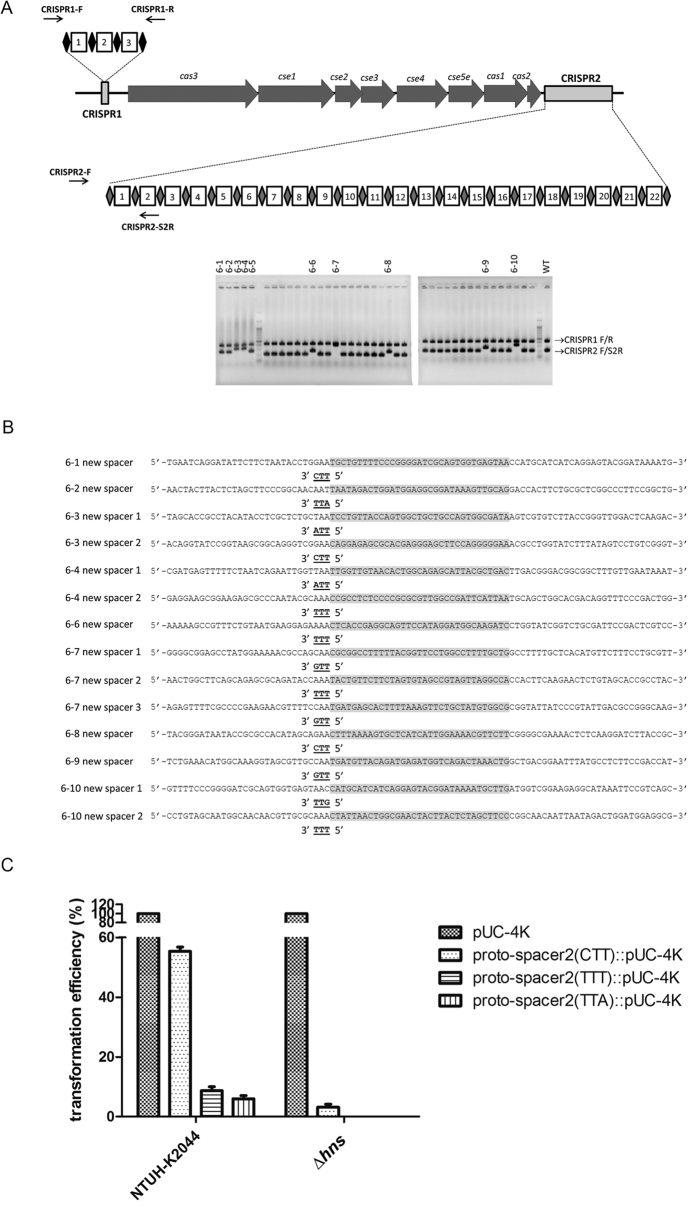
Acquisition of new spacers and PAM sequences in *K. pneumoniae*. The acquisitions of new spacers into CRISPR1 and CRISPR2 arrays were detected by PCR simultaneously using primers (CRISPR1-F and CRISPR1-R for CRISPR1; CRISPR2-F and CRISPR2-S2R for CRISPR2) indicated by the arrows (Fig. 4A). Ten ∆*hns* clones (6-1~6-10) loss of pUC-4K plasmid were detected to have elongated CRISPR2, whereas wild type strain served as a control. Fourteen proto-spacers (marked in gray) and adjacent sequences (upstream and downstream 30 bps) on pUC-4K plasmid were aligned (Fig. 4B). The possible PAM sequences were showed in bold and underlined. The transformation efficiencies of pUC-4K and pUC-4K with engineered proto-spacer 2 carrying different PAM sequences were compared in NTUH-K2044 wild type and *hns* deletion (∆*hns*) (Fig. 4C). The transformation efficiency of pUC-4K was set as 100% and those of proto-spacer2::pUC-4K with different PAM sequences were calculated accordingly. Data are presented as means ± SEM from three independent experiments.

The CRISPR-Cas system was reported to decrease plasmid stability^5^. The stability of a pUC-4K plasmid (with kanamycin resistance) transformed in NTUH-K2044 ∆*hns* mutant (having de-repressed expression of CRISPR-Cas) was examined. Ten out of two hundred clones became sensitive to kanamycin and were confirmed to have lost the pUC-4K plasmid, after six passages of the bacteria in LB broth without kanamycin. New spacers in the CRISPR2 array were found to have been acquired by three clones that had lost the plasmid (Fig. 4A). Sequences of new spacers were identical to the sequences of pUC-4K plasmid. After six passages of ∆*hns*∆*cas3* mutant carrying a pUC-4K plasmid in LB broth, none of 200 clones was sensitive to kanamycin. These results demonstrated that CRISPR-Cas expression could decrease plasmid stability in *K. pneumoniae*.

### PAM sequences analysis

PAM sequences in *K. pneumoniae* were identified by first determining the sequences of newly acquired spacers. The CRISPR2 sequences in 7 clones out of another 39 ∆*hns* mutant clones that had lost the pUC-4K plasmid were observed to be elongated (Fig. 4A). The sequences of newly acquired spacers in ten clones (three clones described above and seven clones here) were analyzed. Each clone had acquired one to three spacers, and the new spacers of two clones (6-2 and 6-5) were identical. In all, 14 new spacers had been identified. Their proto-spacers and adjacent sequences on the pUC-4K plasmid were aligned and analyzed. The only PAM sequence identified in *K. pneumoniae* NTUH-K2044 (12/14, 86%) was 5′-TTN-3′ (Fig. 4B).

The transformation efficiencies of pUC-4K plasmid into ten clones of ∆*hns* mutant harboring pUC-4K plasmid-related spacers (6-1~6-10) were determined as <1 × 10^−9^, while those to ∆*hns* mutant were 3 × 10^−5^, again demonstrating the role of CRISPR-Cas in plasmid transformation.

Spacer sequences identical with spacer 2 in the NTUH-K2044 CRISPR2 flanked by our newly identified PAM sequence (5′-TTT-3′ or 5′-TTA-3′) were further cloned into a pUC-4K plasmid. The transformation efficiencies of pUC-4K plasmid containing proto-spacers carrying different PAM sequences (5′-CTT-3′ or 5′-TTT-3′ or 5′-TTA-3′) were compared in the wild type and ∆*hns* mutant strain (Fig. 4C). The level of interference conferred by proto-spacers carrying 5′-TTT-3′ and 5′-TTA-3′ was greater than that conferred by proto-spacers carrying 5′-CTT-3′. In the ∆*hns* mutant, no transformant was obtained after electroporation with pUC-4K plasmids with proto-spacers carrying 5′-TTT-3′ and 5′-TTA-3′ (<1 × 10^−9^). These results demonstrated that 5′-TTN-3′ was the preferred PAM sequence in *K. pneumoniae*.

### The function of CRISPR1 array on efficiency of plasmid transformation

**Figure 5:**
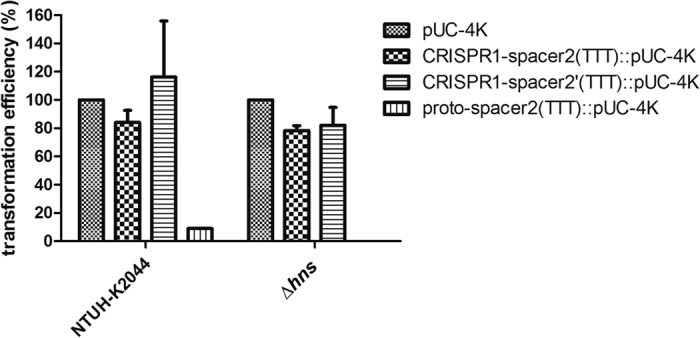
The transformation efficiencies of pUC-4K and pUC-4K with engineered proto-spacer identical with spacer 2 of CRISPR1 in NTUH-K2044 wild type and *hns* deletion (∆*hns*) strain. The transformation efficiency of pUC-4K was set as 100% and those of CRISPR1-spacer2(TTT)::pUC-4K and CRISPR1-spacer2′(TTT)::pUC-4K were calculated accordingly. The transformation of proto-spacer2(TTT) served as a positive control. Data are presented as means ± SEM from three independent experiments.

As shown in Fig. 4A, naturally acquired new spacers were all integrated into the CRISPR2 array. Therefore, CRISPR1 seems to be non-functional for adaptation. To examine the function of CRISPR1 array in interference of plasmid transformation, proto-spacer sequences identical with spacer 2 in the CRISPR1 flanked by PAM sequence (5′-TTT-3′) were further cloned into a pUC-4K plasmid. Then, the transformation efficiencies of plasmids with or without proto-spacer sequences were compared both in wild type and ∆*hns* mutant strain (Fig. 5). In contrast to significant interference conferred by proto-spacer2 which was targeted by crRNA transcribed from CRISPR2 array (as described in Fig. 4C), no interference was detected in transformation of plasmids bearing proto-spacers [CRISPR1-spacer2(TTT) and CRISPR1-spacer2′(TTT)] which were targeted by two-directional crRNA transcribed from CRISPR1 (Fig. 5). Therefore, CRISPR1 seems to be also non-functional for interference.

### Expression of *hns* and CRISPR-Cas system under imipenem treatment

**Figure 6:**
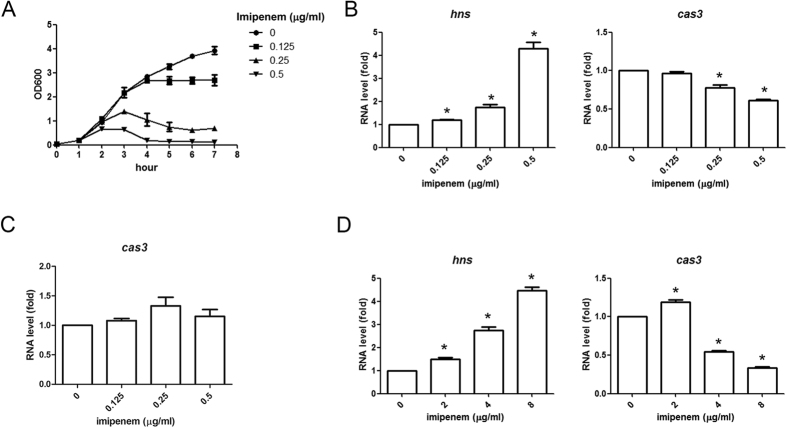
Expression of *hns* and *cas3* under imipenem treatment. The growth curves of NTUH-K2044 strain under different concentrations of imipenem (Fig. 6A). The RNA levels of *hns* and *cas3* treated with different imipenem concentrations in NTUH-K2044 (Fig. 6B), ∆*hns* strain (Fig. 6C) and imipenem resistant N308 strain (Fig. 6D). The RNA expressions of *hns* and *cas3* were determined by quantitative RT-PCR after treatment with different concentrations of imipenem for 3 hours; meanwhile the bacterial growth was determined by absorbance measurement under 600 nm. The RNA level without treatment was set as 1, and those with imipenem treatment were calculated accordingly. Data are presented as means ± SEM from three independent experiments. *P-values of <0.05 were considered significant (Student’s *t*-test).

CRISPR-Cas activity in *K. pneumoniae* NTUH-K2044 strain was demonstrated to be repressed by H-NS. H-NS is considered a global regulator of gene expression in response to environmental stimuli; hence, whether imipenem acts through regulation of H-NS to alter the expression of CRISPR-Cas was further examined. The RNA expressions of *hns* and *cas3* were determined by quantitative RT-PCR after treatment with different concentrations of imipenem (0, 0.125, 0.25 and 0.5 μg/ml) for 3 hours. The bacterial growth curves under different concentrations of imipenem shown in Fig. 6A revealed that the bacterial growth was suppressed after treatment with 0.25 and 0.5 μg/ml of imipenem for 3 hours. The bacterial morphology under microscopic examination was not significantly affected after treatment with imipenem for 3 hours (data not shown). Imipenem induced *hns* RNA expression and inhibited *cas3* RNA expression in NTUH-K2044 wild type strain (Fig. 6B), whereas the *cas3* RNA expression was not altered under imipenem treatment in ∆*hns* mutant (Fig. 6C). These results suggested that imipenem increases *hns* expression and that the induced H-NS subsequently decrease *cas3* expression.

The imipenem effect on *hns* expression was further examined in an imipenem resistant strain N308. The RNA expressions of *hns* and *cas3* were determined by quantitative RT-PCR after treatment with different concentrations of imipenem (0, 2, 4 and 8 μg/ml) for 3 hours. The expression of *hns* was also induced and expression of *cas3* was inhibited in N308 strain after treatment with 4 and 8 μg/ml of imipenem which resulted in suppression of bacterial growth. (Fig. 6D).

### CRISPR-Cas activity under imipenem treatment

To study whether imipenem treatment indeed represses the activity of CRISPR-Cas, we first determined the basal activity of CRISPR-Cas in NTUH-K2044. The 6-1 spacer sequences were cloned into the CRISPR2 array of the wild type strain and then the plasmid transformation efficiencies were compared among strains NTUH-K2044, NTUH-K2044 (6-1 spacer), and ∆*hns* mutant (6-1 spacer). The efficiency of plasmid transformation was low (<1 × 10^−9^) in the ∆*hns* mutant (6-1 spacer) strain with de-repressed expression of CRISPR-Cas, intermediate (7.7 × 10^−7^) in the NTUH-K2044 (6-1 spacer) strain, indicating interference, and high (1.2 × 10^−5^) in the NTUH-K2044 strain. The efficiency of pBK-CMV plasmid transformation (the control) was similar among NTUH-K2044, NTUH-K2044 (6-1 spacer) and ∆*hns* mutant (6-1 spacer) (2.0 × 10^−6^, 2.3 × 10^−6^, and 1.9 × 10^−6^). Collectively, these results reconfirm the basal activity of CRISPR-Cas in NTUH-K2044 wild type.

To confirm the observation of regulation of *hns* RNA and *cas3* RNA expression by imipenem treatment, pUC-4K plasmid stabilities in NTUH-K2044 and NTUH-K2044 (6-1 spacer) was examined with or without imipenem treatment. The pUC-4K plasmid was lost in 63/300 (21%) NTUH-K2044 (6-1 spacer) clones subcultured in LB broth for 8 hours. The loss was significantly decreased (14/300, 4.7%) by the addition of 0.5 μg/ml of imipenem to the subculture, whereas all 300 clones of NTUH-K2044 with or without imipenem treatment were still plasmid-containing. These results indicated the CRISPR-Cas-mediated interference is repressed by imipenem and suggest that exposure of *K. pneumoniae* to imipenem inhibits CRISPR-Cas activity thereby giving *K. pneumoniae* a greater opportunity to acquire resistant genes.

## Discussion

A recent study surveyed CRISPR-Cas systems in *K. pneumoniae* genomes^18^. CRISPR-Cas systems were detected using bioinformatics tools in only 6 out of 52 complete and draft genomes of *K. pneumoniae*; therefore, the CRISPR-Cas system is not widely distributed in *K. pneumoniae*. Blast search analysis showed that 33% (38/116) of spacer sequences were very similar to plasmid, phage, or bacterial genome sequences. In this study, anti-plasmid immunity in *K. pneumoniae* NTUH-K2044 strain was directly attributable to CRISPR-Cas activity. Moreover, the regulation of CRISPR-Cas systems and their correlation with antibiotic resistance in *K. pneumoniae* were also revealed.

H-NS has been shown to repress the CRISPR-Cas system in *E. coli* through direct binding to the promoter of the *cas* operon^14^. In contrast to endogenous expression of CRISPR-Cas in *E. coli* carrying an engineered spacer identical to the corresponding phage lambda sequence, which provided only weak protection against phage infection, disruption of the *hns* gene conferred a high level of protection^13^. These findings were in line with our results in *K. pneumoniae*. As shown in Figs 2 and 4C, the activity of CRISPR-Cas in *K. pneumoniae* NTUH-K2044 strain is not cryptic, but interference with plasmid transformation was prominent in ∆*hns* mutant. The repression of *cas3* expression by *hns* was also confirmed by quantitative RT-PCR. Besides, the acquisition of new spacers (adaptation stage of CRISPR-Cas) was also observed only in those ∆*hns* mutant clones that had lost their plasmids under non-selective conditions. Therefore, the regulation of CRISPR-Cas activity seems to be conserved between *E. coli* and *K. pneumoniae*.

The role of CRISPR-Cas in phage resistance was first described in *Streptococcus thermophilus*^3^. Despite numerous attempts, we failed to isolate ∆*hns* mutant clones resistant to phage NTUH-K2044-K1-1 due to the acquisition of new spacers and their incorporation into CRISPR arrays. Our previous study indicated that components of the capsule may be receptors of phage NTUH-K2044-K1-1^17^, which would explain why most resistant clones were non-encapsulated and lacked detectable CRISPR-Cas activity. Therefore, the function of CRISPR-Cas in the phage resistance of *K. pneumoniae* should be investigated further using other phage strains.

Even though two CRISPR arrays (CRISPR1 and CRISPR2) were detected in *K. pneumoniae* NTUH-K2044 strain, naturally acquired new spacers were all integrated into the CRISPR2 array. Moreover, there was no decrease detected in plasmid transformation conferred by CRISPR1. Therefore, CRISPR1 appears to be non-functional for both adaptation and interference. There were five variable base-pairs revealed in the repeat sequences of CRISPR1 array, which might be the reason contributed to the defect of CRISPR1.

In our study, 5′-TTN-3′ was identified as the preferred PAM sequence in *K. pneumoniae* and differed from the preferred PAM sequence (5′-CTT-3′) in the best-studied type I-E CRISPR-Cas system of *E. coli*^11^,^12^. Two non-consensus PAM sequences were observed (5′-ATT-3′ and 5′-GTT-3′), but the ∆*hns* (6–10 spacer) strain carrying a spacer with 5′-GTT-3′ also had another spacer bearing the consensus PAM sequence. A previous study reported multiple spacers integrated into a single *E. coli* clone, all targeting the same DNA strand^11^. However, an exception was observed in our study, that is, a ∆*hns* (6–10 spacer) strain carrying two spacers targeting different strands. Therefore, the mechanism of adaptation still awaits further study.

Inverse correlation between the presence of CRISPR-Cas loci and carbapenem resistance was also revealed in *K. pneumoniae* in this study. The function of *K. pneumoniae* CRISPR-Cas in adaptation and interference was fully demonstrated in the absence of H-NS, confirming activated CRISPR-Cas indeed could prevent the assimilation of foreign DNA such as antibiotic resistance genes. Researchers hypothesized that H-NS will bind to invading AT-rich DNA, then consequently free the *cas* promoter, thus bolstering the defense against foreign DNA by CRISPR-Cas^19^. Otherwise, the exchange of DNA facilitates the rapid adaptation of bacteria to environmental change. The gaining of new genetic material needs to be delicately balanced against the limiting of horizontal gene transfer. In this study, we observed that imipenem treatment caused decreased CRISPR-Cas activity by induced H-NS expression. Through this regulatory mechanism, bacteria might become more able to acquire resistance genes to confront the challenge of antibiotics.

The imipenem effect on *hns* expression level was observed in both imipenem sensitive and resistant strains when encountered suppression of growth. Previous study also demonstrated the expression of *hns* was induced under cold-shock stress^20^. Therefore, we suggested that unrelated antibiotic or stress might have the same effect on *hns* expression level. We also examined the imipenem effect on transformation efficiency. However, electroporation caused approximately 100-fold bacterial deaths in imipenem-treated bacteria than in untreated bacteria. The transformation efficiency should be better analyzed by natural transformation rather than by electroporation. However, ability of natural transformation was not observed in our strain.

Besides involvement in defense against foreign DNA, CRISPR-Cas has been correlated with bacterial virulence^21^. A recent study indicated that the type II CRISPR-Cas system of *Legionella pneumophila* has an important role in intracellular survival and replication in amoebae^22^. The type I-F CRISPR-Cas system of *Pseudomonas aeruginosa* has been demonstrated to be involved in biofilm formation and swarming^23^. Cas9 of the type II-B CRISPR-Cas system from *Francisella novicida* has been shown to repress the expression of bacterial lipoprotein (BLP) and thereby contributes to immune avoidance during infection^24^. Therefore, whether the CRISPR-Cas of *K. pneumoniae* has other roles such as bacterial virulence needs further study.

In conclusion, NTUH-K2044 CRISPR-Cas contributes to decrease of plasmid transformation and plasmid stability. The basal activity of CRISPR-Cas can be repressed through induction of H-NS by imipenem.

## Methods

### Bacterial strains

The *K. pneumoniae* NTUH-K2044 strain causing pyogenic liver abscess and meningitis^25^, and 85 carbapenem-resistant *K. pneumoniae* (CRKP) strains obtained from four hospitals located in north or south Taiwan as described elsewhere were used in this study^26^. A total of 132 drug-sensitive intestinal strains were collected for comparison. Stool specimens were collected from healthy volunteers who had health checkups in the Health Management Center of National Taiwan University Hospital during May to November 2006. The stool specimens were collected in fecal occult blood test tubes, stored at 4 °C, cultured on EMB agar plates, and identified as *K. pneumoniae* using the Enterotube system (BD, NJ, USA). This study protocol was approved by the Institutional Review Board of National Taiwan University Hospital (IRB approval number: 9561701018). The methods were carried out in accordance with the approved guidelines and written informed consent was obtained from each participant.

Both *K. pneumoniae* and *E. coli* were grown in Luria-Bertani (LB) broth or agar at 37 °C, except as noted below. Where appropriate, medium was supplemented with kanamycin (50 μg/mL) or sucrose (5%).

### Analysis of CRISPR-Cas sequences

The CRISPR-Cas sequences were analyzed by using CRISPRFinder on the CRISPRs web server (http://crispr.u-psud.fr/Server/).

### Prevalence of CRISPR-Cas

The prevalence of CRKP strains and intestinal strains with CRISPR-Cas was determined by PCR using primers (cas3-F1 5′-TGGCCGACATTTGATTCAGC-3′ and cas3-R1 5′-CCATGCTTAACATTCATCAC-3′ for *cas3*; CRISPR1-F 5′-GACGGTGGTTATATGGTGAC-3′ and CRISPR1-R 5′-CATTGATGCCTCTACGTCAG-3′ for CRISPR1; CRISPR2-F 5′-GATCTCAGTGGGTTACAGC-3′ and CRISPR2-R 5′-CCAAACGACAGTTTCATTAG-3′ for CRISPR2).

### Construction of ∆*hns* and ∆*cas3* deletion mutants

The *hns (kp3314*) and *cas3 (kp3171*) deletion mutants were constructed as follows. The genes and its flanking regions were amplified by using primers 5′-GGTCGACTTACCTGCATTC-3′ and 5′-CTCGCTGAGATGATCTCTC-3′ for *hns*; 5′-CTGCAATAACGACGTCAGTTC-3′ and 5′-GTTTATGGGCAGCAATAACCG-3′ for *cas3* and then cloned into a pGEM-T easy plasmid (Promega, WI, USA). The deleted fragment was generated by inverse PCR using primers 5′-TGTAGTAATCTCAAACTTA-3′ and 5′-TCTCCGTTGATCGCTATAA-3′ for *hns*; 5′-ACCATGGAGAACCGCTTCAAT-3′ and 5′-GGAATTTTTCCTTAAAAAACATGTG-3′ for *cas3* and then subcloned into a NotI site of pKO3-km plasmid. The resulting constructs were electroporated into wild type NTUH-K2044 strain. The deletion mutants were selected as previously described^27^ and confirmed by PCR as well as sequencing with appropriate primers.

### Construction of *hns* complementation strain

The *hns* and its putative promoter were PCR amplified (5′-GGTCGACTTACCTGCATTC-3′ and 5′-TTAGATCAGGAAATCGTCCAG-3′) and cloned into the intergenic region of the two open reading frames, *pgpA* and *yajO,* in a pKO3-Km-*pgpAyajO* recombinant vector^28^. The resulting construct was electroporated into a ∆*hns* mutant strain. The complementation strain was selected as previously described^28^ and confirmed by PCR as well as sequencing with appropriate primers.

### Construction of plasmids carrying engineered proto-spacer

The engineered proto-spacer2 fragments carrying spacer2 of CRISPR2 and different proto-spacer adjacent motif (PAM) sequences were synthesized by annealing single-stranded, complementary oligonucleotides and then cloned into a ScaI site of pUC-4K plasmid (5′-AAGCACCACGATCTCTATCACCGACGCGCCGACTAC-3′ and 5′-GTAGTCGGCGCGTCGGTGATAGAGATCGTGGTGCTT-3′ for that with PAM sequences 5′-CTT-3′; 5′-NAACACCACGATCTCTATCACCGACGCGCCGACTAC-3′ and 5′-GTAGTCGGCGCGTCGGTGATAGAGATCGTGGTGTTN-3′ for that with PAM sequences 5′-TTN-3′). The resulting plasmids were confirmed by sequencing.

The proto-spacers [CRISPR1-spacer2(TTT) and CRISPR1-spacer2′(TTT)] identical with spacer 2 in the CRISPR1 flanked by PAM sequence (5′-TTT-3′) which were targeted by two-directional crRNA transcribed from CRISPR1 were cloned into pUC-4K plasmid as described above (5′-AAACTATTTCGGGTCCAACAAACGGCACGCCGATC-3′ and 5′-GATCGGCGTGCCGTTTGTTGGACCCGAAATAGTTT-3′ for CRISPR1-spacer2(TTT); 5′-AAAGATCGGCGTGCCGTTTGTTGGACCCGAAATAG-3′ and 5′-CTATTTCGGGTCCAACAAACGGCACGCCGATCTTT-3′ for CRISPR1-spacer2′(TTT)).

### Transformation efficiency

Aliquots of approximately 1 × 10^9^ cfu of *K. pneumoniae* bacteria in 100 μl of 10% glycerol were mixed with 1 μg of plasmid DNA, and then shocked by using an electroporator (BTX ECM630, MA, USA) with settings as below (Voltage = 2500 V, Resistance = 200 ohms, Capacitance = 50 μf). After recovery in LB broth for one hour, the total and transformed bacterial numbers were enumerated by plating after serial dilutions on LB and LB supplemented with kanamycin plates, respectively. The transformation efficiency was calculated accordingly.

### Reverse transcription quantitative polymerase chain reaction (RT-qPCR)

The RNAs were extracted by using an RNeasy mini kit per manufacturer’s instructions (Qiagen, Hilden, Germany). A total of 400 ng total RNA was used as template for RT. The RNA expression levels were measured by quantitative PCR using an ABI 7900 thermocycler. The primers for *hns* (5′-CGCGGCAGAAATTGAAGAG-3′ and 5′-AGCCATGGTGCTCAGCAGTT-3′), *cas3* (5′-TTTCCCCATTCCCATTTGC-3′ and 5′-CGATCCACCGAAGAAACCA-3′) and 23S ribosomal RNA internal control (5′-GGTTAAGCGACTAAGCGTACACGGT-3′ and 5′-ACGAGGCGCTACCTAAATAGCTTTC-3′) were used. The relative RNA expression was calculated according to the ∆∆Ct value.

### Plasmid stability

NTUH-K2044 and ∆*hns* mutant transformed with pUC-4K plasmid was used to inoculate 5 ml of LB broth. Fifty microliters of the previous culture was inoculated into 5 ml of fresh LB medium every morning (37 °C) and night (30 °C) for six passages. For each culture, bacteria were plated on LB plates and kanamycin-sensitive colonies were screened after replica on LB plates with kanamycin. The CRISPR1 and CRISPR2 of the kanamycin-sensitive clones were detected by PCR (CRISPR1-F and CRISPR1-R for CRISPR1; CRISPR2-F and CRISPR2-S2R 5′-GTGATAGAGATCGTGGTG-3′ for CRISPR2) and sequencing.

### Construction of NTUH-K2044 (6-1 spacer)

The CRISPR2 array carrying the 6-1 spacer sequences was amplified from ∆*hns* mutant (6-1 spacer) by using primers (5′-GCTTTATCCATTCAGGTAG-3′ and 5′-CAGCCAATTTGTAACCTGTG-3′) and then cloned into a pKO3-km plasmid. The resulting plasmid was electroporated into wild type strain. The strain with insertion of 6-1 spacer was selected as previously described^27^ and confirmed by PCR as well as sequencing with appropriate primers.

### Statistical analysis

Data are presented as means ± standard error of the mean (SEM) from three independent experiments. Statistical significance was assessed by a two-tailed Student’s *t*-test or chi-square test using Prism5 (GraphPad Prism) software. *P-*values of <0.05 were considered significant.

## Additional Information

**How to cite this article**: Lin, T.-L. *et al*. Imipenem represses CRISPR-Cas interference of DNA acquisition through H-NS stimulation in *Klebsiella pneumoniae. Sci. Rep.* **6**, 31644; doi: 10.1038/srep31644 (2016).
